# Efficacy and safety of TNF-α inhibitors for active ankylosing spondylitis patients: Multiple treatment comparisons in a network meta-analysis

**DOI:** 10.1038/srep32768

**Published:** 2016-09-26

**Authors:** Wei Liu, Yuan-hao Wu, Lei Zhang, Xiao-ya Liu, B X Bin Xue, B L Bin Liu, Yi Wang, Yang Ji

**Affiliations:** 1 Department of Rheumatology and Immunology, First Teaching Hospital of Tianjin University of Traditional Chinese Medicine, Tianjin, China; 2 Institute of Basic Research in Clinical Medicine, China Academy of Chinese Medical Science, Beijing 100700, P. R. China; 3 The 272nd Hospital of Chinese People’s Liberation Army, Tianjin, China

## Abstract

Ankylosing spondylitis (AS) is an inflammatory rheumatic disease with impact on axial skeleton, peripheral joints and enthuses, and it may result in severe disabilities of those parts. Tumor necrosis factor-α (TNF-α) inhibitors are considered as an effective treatment for patients with active AS. In this study, we conducted a network meta-analysis to compare the clinical outcomes of active AS patients treated with TNF-α inhibitors. Randomized controlled trials (RCTs) evaluating the efficacy and safety of TNF-α inhibitors were retrieved in literature search and selected for meta-analysis. Changes in ASAS20 response, ASAS40 response and BASDAI 50% response were regarded as efficacy outcomes; serious adverse events (SAE) and all cause withdrawals were regarded as safety outcomes. Both traditional pairwise meta-analysis and network meta-analysis were performed. The results showed that adalimumab and infliximab had better clinical outcomes. Infliximab consistently appeared to be the most effective TNF-α inhibitors with a high risk of adverse events for patients with active AS; meanwhile, adalimumab ranked highest with respect to adverse effects with efficacy secondary to infliximab. As a result, we were unable to conclude the optimal TNF-α inhibitor and this issue should be solved by future researchers.

Ankylosing spondylitis (AS) is an inflammatory rheumatic disease which affects the axial skeleton, peripheral joints and enthuses. It is characterized by inflammatory back pain, which not only leads to both structural and functional disability but also affects one’s life quality^1^. A large proportion of AS patients are young adults with males roughly two times as likely as females to suffer from this disease^2^. The overall annual incidence of AS is 0.5–14 per 100,000 people over the world^1^ and its prevalence in Caucasian is estimated to be approximately 0.5%^3^. The inflammatory back pain of AS is featured by worsen stiffness and pain usually occurs in the morning after long periods of inactivity. Although this kind of pain is not able to be relieved by taking a rest, it can be significantly improved with exercise and non-steroidal anti-inflammatory drug (NSAID). It is established that AS is associated with Human leukocyte antigen B27 (HLA-B27) and individuals with certain subtypes of HLA-B27 are more susceptible to AS^4^. The modified New York Classification Criteria^5^ is widely used for diagnosis and classification of AS^2^.

AS can be controlled by relieving symptoms (pain, stiffness, joint swelling), improving physical function, and delaying or avoiding structural damages, which can result in physical impairments and deformities^6^. The European League against Rheumatism (EULAR) and the Assessments in Ankylosing Spondylitis International Society (ASAS) recommended that NSAIDs, biological agents, disease modifying antirheumatic drugs (DMARDs), analgesics, steroids, non-pharmacological treatment (including education, exercise, and physiotherapy) and surgical interventions can be introduced to relieve AS symptoms^7^. Furthermore, TNF-α inhibitors have been verified as a significant breakthrough for managing patients with active AS and they are able to relieve symptoms caused by AS in a rapid manner for the majority of patients. Besides that, TNF-α inhibitors can normalize acute phase reactants and reduce acute inflammation seen in SI joints and spines^8^.

Currently, TNF-α inhibitors in common use include adalimumab, etanercept, golimumab and infliximab. Although the efficacy and safety of each TNF-α inhibitor have been assessed in clinical trials, there appeared to be considerable variation with respect to the efficacy and safety indicators in the literatures. In order to provide concrete evidence for clinical practice, it is of great importance to perform a thorough comparison of all relevant TNF-α inhibitors. This study enabled us to achieve this objective through implementation of a network meta-analysis which takes adalimumab, etanercept, golimumab and infliximab into account.

## Material and Methods

### Literature search and selection criteria

PubMed, EMBASE, the Cochrane and Scopus Database were searched for published RCTs investigating the efficacy of TNF-α inhibitors for AS patients from January 2001 to August 2015. Key terms including “TNF-α inhibitors”, “ankylosing spondylitis” and “randomized controlled trials” were used with no other restrictions. The detailed research strategy of each database was shown in Table S1. References of all retrieved publications were also searched manually for relevant studies. Two reviewers evaluated the qualification of retrieved publications independently. The systematic review was performed in accordance with PRISMA (Preferred Reporting Items for Systematic Reviews and Meta-Analyses) guidelines^9^.

RCTs were eligible for this study if the efficacy or safety is evaluated between a TNF-α inhibitor and another TNF-α inhibitor (or placebo) for active AS patients management. Trials were included into the network meta-analysis if they met the following criteria: (i) trials were randomized-design and patients were over 18 years of age; (ii) AS was diagnosed based on the 1984 modified New York criteria^10^; (iii) trials reported relevant efficacy outcomes or measures of variance for the outcomes. Trials were excluded if they were (i) trials of axial spondyloarthritis without subgroup analysis of AS patients; (ii) trials where patients had concomitant use of TNF-α inhibitors^11^.

### Data extraction and assessment of bias

Data was independently extracted by two authors. Study design, characteristics of participants, treatment of pharmaceuticals, follow-up time and relevant outcomes were documented. Any disagreement regarding to data extraction was determined by a third investigator. For cross-over trials, if a washout period was presented before the cross-over time and results of the two phases were reported separately, the results of the second phase were recorded as a separate study. In this study, we analyzed the changes in ASAS20 response, ASAS40 response and BASDAI 50% response as efficacy outcomes; serious adverse events (SAE) and all cause withdrawals were considered as safety outcomes.

The Jadad Scale was used for quality assessment of case-control studies in meta-analysis. A paper reporting a clinical trial could therefore receive a Jadad score of between zero and five; scores equal to or above 3 were considered as of high quality.

### Statistical analysis

First, traditional meta-analyses were conducted on studies directly comparing one TNF-α inhibitor with a placebo. Then, Bayesian network meta-analyses were performed to compare over different TNF-α inhibitors.

In traditional meta-analyses, odd ratios (ORs) with their corresponding 95% CIs were used to assess the efficacy or safety of TNF-α inhibitors. Heterogeneity was examined using the Cochran’s *Q*-statistic and a *P*-value of less than 0.01 was considered significant. The *I*^*2*^ test was also used to quantify heterogeneity (ranging from 0 to 100%). *P* < 0.01 for *Q*-test or *I*^*2*^ > 50% indicated the existence of heterogeneity across the studies. Random-effect model (*DerSimonian-Laird* method) was used in order to minimize the effect of heterogeneity. All statistical analysis in these meta-analyses was conducted using STATA version 12.0 (Stata Corp, College Station, TX, USA).

In addition to traditional meta-analyses, which directly compared two different treatments, network meta-analyses were also performed to form indirect comparisons. The network meta-analyses were performed with a random-effects model based on a Bayesian framework using Markov Chain Monte Carlo methods in WinBUGS (MRC Bio-statistics Unit, Cambridge, UK). A probabilistic analysis was performed to estimate rank probabilities based on network meta-analysis. The rank probabilities were summarized for each drug in order to obtain the surface under the cumulative ranking curve (SUCRA) as previously described^12^. Moreover, we compared pooled odds ratios (ORs) from network meta-analyses and their corresponding ORs with those computed by traditional meta-analyses to assess the consistence between direct and indirect evidence. The inconsistency was estimated by the node-splitting method which generates *P* values for the null hypothesis that there is no significant inconsistency between direct and indirect evidence.

## Results

### Study characteristics

A total of 1,855 studies were retrieved from databases and relevant articles. Sixteen studies^13^,^14^,^15^,^16^,^17^,^18^,^19^,^20^,^21^,^22^,^23^,^24^,^25^,^26^,^27^,^28^ were eligible for the analyses after further screening, with a total of 2,574 AS patients involved (Table 1). Efficacy (ASAS20 response, ASAS40 response and BASDAI 50% response) and safety (SAE and all cause withdrawals) of four TNF-α inhibitors, adalimumab, etanercept, golimumab and infliximab, were analyzed in this study. The bias assessment was presented in Table S2, indicating that all included studies were of high quality.

### Results from traditional meta-analyses

In traditional meta-analyses, we obtained direct evidence by comparing four TNF-α inhibitors with placebo (Table 2). As suggested by random effects model for responses including ASAS20, ASAS40 and BASDAI 50%, adalimumab, etanercept, golimumab and infliximab were significantly more effective than placebo (OR > 1) (Supplement Figures 1–3). There was no significant difference in safety between TNF-α inhibitors and placebo (*P* > 0.05) (Supplement Figures 4 and 5).

### Results from network meta-analyses

**Figure 1:**
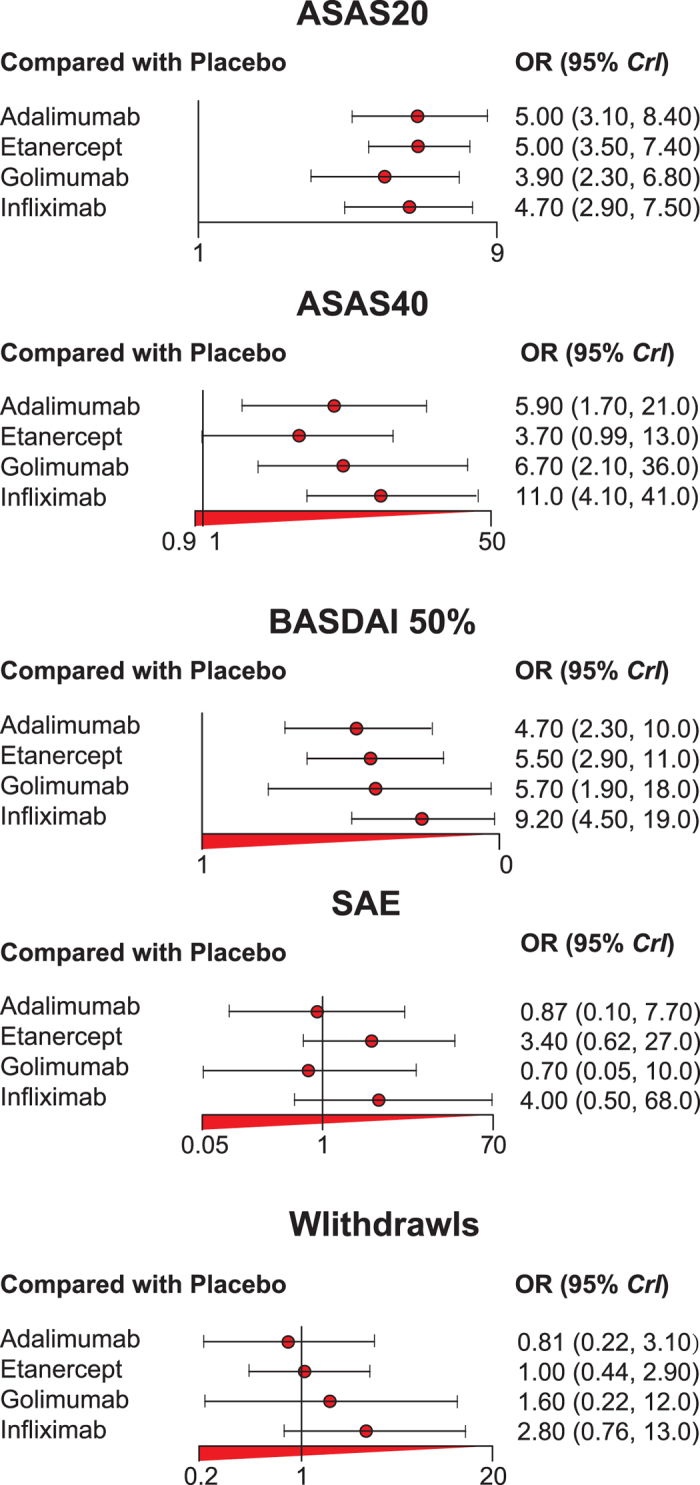
Forest plots of four TNF-α inhibitors for five clinical outcomes. The OR values from each study are represented by squares, and the credible intervals (CrIs) are indicated by error bars. The rhombus indicates whether the pooled OR value is under the random effects model.

We carried out comparisons among these four inhibitors through network meta-analysis. ORs and their corresponding 95% credible intervals (CrIs) from network meta-analyses were presented in Fig. 1 and Table 3. We compared the efficacy and safety of adalimumab, etanercept, golimumab, infliximab and placebo by synthesizing both direct and indirect comparisons. The results were in compliance with traditional meta-analyses. Significant improvement in clinical responses (OR > 1) and insignificant difference in adverse events (*P* < 0.05) were observed from network meta-analyses. Adalimumab, etanercept, golimumab, infliximab showed significant difference comparing to placebo in both ASAS20 and BASDAI 50%. Etanercept did not prove its significant efficacy in ASAS40. It is estimated that all inhibitors had no significant variation to placebo in safety issues.

**Figure 2:**
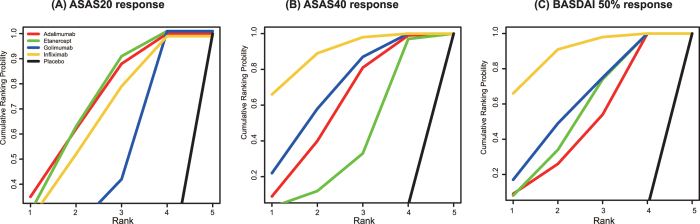
Plots of the SUCRA probabilities for ASAS20, ASAS40 and BASDAI 50% response. (**A**) ASAS20 response; (**B**) ASAS40 response and (**C**) BASDAI 50% response. The area under the curve is equivalent to the value of SUCRA, and thus a bigger area corresponds to a better outcome.

**Figure 3:**
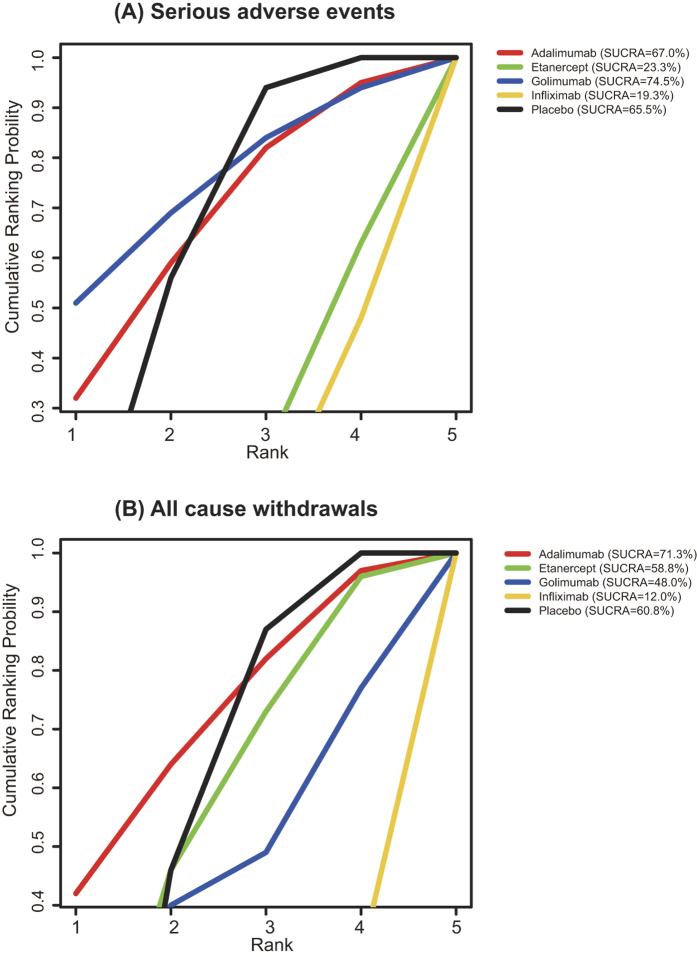
Plots of the SUCRA probabilities for serious adverse events and all cause withdrawals. (**A**) Serious adverse events; (**B**) all cause withdrawals. The area under the curve is equivalent to the value of SUCRA, and thus a bigger area corresponds to a better outcome.

In secondary analyses, we compared the estimated rank probabilities of different pharmaceutics concerning various outcomes. Adalimumab had the highest efficacy in ASAS20 response, whereas infliximab had higher rank probabilities in both ASAS40 and BASDAI 50% responses (Fig. 2). Golimumab had the lowest probability of SAE (Fig. 3A), whereas drug withdrawals were least likely to occur in patients treated with adalimumab (Fig. 3B). Lastly, infliximab had the worst performance with respect to SAE and all cause withdrawals.

### Comparisons between traditional pairwise and network meta-analyses

Results from traditional and network meta-analyses were presented in Tables 2 and 3. The results were generally consistent, with insignificant differences in OR values and corresponding 95% CI. Since closed loop was not presented in the stellate data, the node-splitting method could not be performed. Furthermore, the relative ranking of four drugs based on their SUCRA were shown in Table 4.

## Discussion

In this meta-analysis, we reviewed the clinical outcomes of AS patients treated with various TNF-α inhibitors. The corresponding results showed that four TNF-α inhibitors could significantly alleviate clinical symptoms of AS. Although indirect and direct comparisons were conducted, no statistical differences were found among adalimumab, infliximab, etanercept and golimumab. Therefore, we were unable to conclude which TNF-α inhibitor had the best performance in terms of both efficacy and safety.

TNF-α is a biological agent which is primarily produced by macrophages and activated monocytes during inflammatory responses. It can also induce the production of other proinflammatory cytokines. Apart from that, TNF-α stimulates endothelial cells in order to express adhesion molecules so that leukocytes can be attracted into inflammatory joints. TNF-α can also promote metallo proteinase synthesis and inhibit the synthesis of proteoglycans in cartilage^29^. Since TNF-α is related to pain, tenderness, swelling and fever caused by many inflammatory conditions, four TNF-α inhibitors including adalimumab (Humira^®^), etanercept (Enbrel^®^), golimumab (Simponi^®^) and infliximab (Remicade^®^) have been developed to target the function of TNF-α and reduce pain, swelling and inflammation of AS patients^30^.

The studied four drugs affect the function of TNF-α via different mechanisms. Adalimumab is a recombinant human IgG1 monoclonal antibody specific to human TNF-α^31^, while golimumab is a human monoclonal antibody binding to both soluble and transmembrane TNF-α^32^. Etanercept is a receptor fusion protein which specifically binds to TNF-α receptors and inhibits the binding of TNF-α to cell surface^33^. Infliximab is a chimeric (mouse/human) monoclonal antibody of the IgG1κ isotype that binds to TNF-α with a high affinity^34^. All four agents can prevent TNF-α from promoting inflammation, resulting in its application in effective treatments for AS patients. In this study, pair-wise meta analysis indicated that four TNF-α inhibitors were significantly more effective than placebo with respect to three efficacy outcomes (ASAS20, ASAS40, BASDAI 50% response), while such a trend was not observed in safety outcomes (serious adverse events, all cause withdrawals). Maxwell *et al*. indicated that adalimumab, etanercept, infliximab and golimumab had better performances than the placebo with respect to ASAS40, while no significant difference was found between these inhibitors and placebo when withdrawals and serious adverse events were taken in to account. Results from our study were consistent with those provided by Maxwell *et al*.

Our study provided exclusive evidence for assessing the current available TNF-α inhibitors that are used for managing AS patients. Our network meta-analysis incorporated all comparisons of available TNF-α inhibitors into a single analysis. In addition, a total of 2,574 AS patients were included in our research in order to provide a ranking of these TNF-α inhibitors when both efficacy and adverse effects were taken into consideration. However, there was no significant evidence to conclude the optimal TNF-α inhibitor for managing AS patients.

Nevertheless, there are several perspectives which concern about the limitation of our research due to its nature or design. First of all, variations in treatment duration or dose may have significant impact on the overall outcomes and we are unable to cope with this issue since a large number of randomized clinical trials with different designs were included in our research. Secondly, the number of comparisons or the sample size for each comparison varied significantly and some studies may have an unexpectedly significant impact on the overall effect size while others may not have such an impact. For instance, only two RCTs assessing golimumab were included in our research and this certainly did not provide sufficient evidence. Thirdly, other confounding factors such as treatment delivery methods may have influence on the clinical outcomes of AS patients. For example, different delivery methods of interventions may result in different reasons of withdrawals and thus affect the evaluation of safety outcomes. In this study, we observed that patients treated with intravenous infusion infliximab exhibited the highest rate of withdrawal and we suspected that intervention delivery approaches may be associated with the likelihood of withdrawals.

In summary, the four TNF-α inhibitors have superior clinical performance for managing AS patients when compared with placebo. However, we were unable to conclude the optimal TNF-α inhibitor and this issue should be solved by future researchers.

## Additional Information

**How to cite this article**: Liu, W. *et al*. Efficacy and safety of TNF-α inhibitors for active ankylosing spondylitis patients: Multiple treatment comparisons in a network meta-analysis. *Sci. Rep.* **6**, 32768; doi: 10.1038/srep32768 (2016).

## Supplementary Material

Supplementary Information

## Figures and Tables

**Table 1:** Main characteristics of enrolled studies.

First author, year	Follow-up	Treatment group 1								Treatment group 2						
	(wks)	Treatment	Dose	N	Mean age	Disease duration	BASDAI (0-10)	BASFI (0-10)	BASMI (0-10)	Treatment	N	Mean age	Disease duration	BASDAI (0-10)	BASFI (0-10)	BASMI (0-10)
Bao C, 2014	14	Golimumab	50 mg Q4W	108	30.5	4.2	6.5 (1.3)	5.0 (2.4)	4.0 (1.9)	Placebo	105	30.6	3.7	6.5 (1.5)	5.0 (2.4)	4.0 (1.9)
Brandt J, 2003	6	Etanercept	25 mg BIW	14	39.8	14.9	6.5 (1.2)	6.2 (1.8)	4.1 (1.7)	Placebo	16	32	11.4	6.6 (1.0)	5.3 (2.3)	3.8 (2.1)
Brandt J, 2004	12	Infliximab	5 mg/kg	34	40.6	16.4	6.5 (1.2)	5.4 (1.8)	3.7 (2.0)	Placebo	35	39	14.9	6.3 (1.4)	5.1 (2.2)	3.7 (2.2)
Braun J, 2002	12	Infliximab	5 mg/kg	34	40.6	16.4	6.5 (1.9)	5.6 (1.9)	3.7 (2.1)	Placebo	35	39	14.9	6.4 (1.6)	5.1 (2.1)	3.8 (2.3)
Calin A, 2004	12	Etanercept	25 mg BIW	45	45.3	15	6.1 (0.9)	NA	NA	Placebo	39	40.7	9.7	5.9 (1.3)	NA	NA
Davis JC, 2003	24	Etanercept	25 mg BIW	138	42.1	10.1	5.8 (1.5)	NA	NA	Placebo	139	41.9	10.5	6.0 (1.4)	NA	NA
Dijkmans B, 2009	12	Etanercept	25 mg BIW	43	NA	NA	6.1 (0.6)	6.0 (0.5)	NA	Placebo	39	NA	NA	5.9 (0.5)	5.7 (0.4)	NA
Dougados M, 2011	12	Etanercept	50 mg QW	39	46	19	6.4 (1.2)	6.3 (2.0)	5.7 (1.4)	Placebo	43	48	23	5.8 (1.5)	5.7 (1.9)	5.8 (1.3)
Gorman JD, 2002	16	Etanercept	25 mg BIW	20	38	15	NA	NA	NA	Placebo	20	39	12	NA	NA	NA
Huang F, 2014	12	Adalimumab	40 mg Q2W	229	30.1	8.1	NA	4.3 (2.3)	3.4 (1.4)	Placebo	115	29.6	7.7	NA	4.4 (2.3)	3.4 (1.5)
Inman RD, 2008	14	Golimumab	50 mg Q4W	138	38	11	6.8 (0.5)	5.2 (1.0)	3.0 (0.8)	Placebo	78	41	16	6.5 (0.5)	4.9 (0.8)	4.0 (0.8)
Inman RD, 2010	12	Infliximab	3 mg/kg	39	42.9	11.7	NA	NA	NA	Placebo	37	39.3	11.1	NA	NA	NA
Marzo-Ortega H, 2005	30	Infliximab	5 mg/kg	28	41	8	6.9 (1.8)	6.7 (1.9)	NA	Placebo	14	39	10	6.4 (1.7)	6.0 (1.4)	NA
van der Heijde D, 2005	24	Infliximab	5 mg/kg	201	40	7.7	6.6 (0.6)	5.7 (0.6)	4.0 (0.7)	Placebo	78	41	13.2	6.5 (0.5)	6.0 (0.8)	4.0 (0.8)
van der Heijde D, 2006a	12	Etanercept	25 mg BIW	305	39.8	10	6.2 (1.7)	6.1 (2.0)	NA	Placebo	51	40.1	8.5	6.1 (1.4)	6.0 (1.9)	NA
van der Heijde D, 2006b	12	Adalimumab	40 mg Q2W	208	41.7	11.3	5.2 (2.2)	6.3 (1.7)	3.8 (2.2)	Placebo	107	43.4	10	6.3 (1.7)	5.6 (2.2)	4.2 (2.1)

NA: not available.

**Table 2:** Results of traditional meta-analyses.

Comparisons	Model	ASAS20 response		ASAS40 response		BASDAI 50% response		Serious adverse events		All cause withdrawals	
		N, n	OR [95% CI]	N, n	OR (95% CI)	N, n	OR (95% CI)	N, n	OR (95% CI)	N, n	OR (95% CI)
Adalimumab vs. Placebo	Fixed	2 (437, 222)	**4.98 [3.47–7.15]**	2 (437, 222)	**5.67 [3.59–8.98]**	2 (437, 222)	**4.69 [3.14–7.02]**	2 (437, 222)	0.89 [0.25**–**3.12]	2 (437, 222)	0.78 [0.33**–**1.84]
	Random		**4.98 [3.47–7.15]**		**5.70 [3.35 -9.70]**		**4.69 [3.14–7.02]**		0.89 [0.25**–**3.12]		0.78 [0.33**–**1.84]
Etanercept vs. Placebo	Fixed	7 (604, 347)	**4.81 [3.53–6.55]**	2 (344, 94)	**3.72 [2.11–6.55]**	4 (401, 149)	**5.00 [3.03–8.25]**	5 (522, 265)	2.36 [0.85**–**6.60]	6 (559, 308)	0.99 [0.55**–**1.79]
	Random		**4.81 [3.53–6.55]**		**3.72 [2.11–6.55]**		**5.00 [3.03–8.28]**		2.36 [0.85**–**6.60]		0.99 [0.55**–**1.79]
Golimumab vs. Placebo	Fixed	2 (246, 183)	**3.82 [2.49–5.87]**	2 (246, 183)	**5.17 [2.70–9.87]**	1 (138, 78)	**5.58 [2.72–11.47]**	1 (138, 78)	0.70 [0.18**–**2.67]	1 (108, 105)	1.49 [0.41**–**5.42]
	Random		**3.87 [2.18–6.85]**		**6.72 [1.84–24.55]**		**5.58 [2.72–11.47]**		0.70 [0.18**–**2.67]		1.49 [0.41**–**5.42]
Infliximab vs. Placebo	Fixed	5 (336, 199)	**4.60 [3.05–6.96]**	3 (274, 150)	**8.89 [4.86–16.27]**	4 (308, 185)	**8.36 [4.84–14.44]**	3 (263, 127)	2.01 [0.49**–**8.23]	3 (274, 150)	1.94 [0.69**–**5.44]
	Random		**4.37 [2.60–7.33]**		**9.94 [4.46–22.14]**		**8.36 [4.70–14.87]**		2.03 [0.49**–**8.51]		1.99 [0.61**–**6.50]

Bold values indicate statistical differences.

**Table 3:** Results of network meta-analysis.

**ASAS20 response**	**Adalimumab**	0.99 (0.53, 1.89)	0.78 (0.37, 1.62)	0.95 (0.46, 1.79)	**0.20 (0.12**, **0.32)**
	1.01 (0.53, 1.89)	**Etanercept**	0.78 (0.41, 1.48)	0.96 (0.53, 1.61)	**0.20 (0.14**, **0.29)**
	1.28 (0.62, 2.71)	1.28 (0.68, 2.43)	**Golimumab**	1.21 (0.57, 2.37)	**0.26 (0.15**, **0.43)**
	1.05 (0.56, 2.17)	1.04 (0.62, 1.88)	0.83 (0.42, 1.75)	**Infliximab**	**0.21 (0.13**, **0.33)**
	**5.00 (3.14**, **8.33)**	**4.98 (3.45**, **7.26)**	**3.89 (2.35**, **6.72)**	**4.69 (3.03**, **7.43)**	**Placebo**
**ASAS40 response**	**Adalimumab**	0.64 (0.09, 3.96)	1.17 (0.21, 10.32)	1.90 (0.35, 13.60)	**0.17 (0.05**, **0.61)**
	1.57 (0.25, 10.92)	**Etanercept**	1.89 (0.33, 17.25)	3.04 (0.56, 22.97)	0.27 (0.07, 1.04)
	0.86 (0.10, 4.73)	0.53 (0.06, 3.02)	**Golimumab**	1.61 (0.22, 10.18)	**0.14 (0.03**, **0.48)**
	0.53 (0.07, 2.84)	0.33 (0.04, 1.78)	0.62 (0.10, 4.56)	**Infliximab**	**0.09 (0.02**, **0.26)**
	**5.89 (1.63**, **21.25)**	3.69 (0.96, 13.48)	**6.94 (2.10**, **37.14)**	**11.23 (3.89**, **45.10)**	**Placebo**
**BASDAI 50% response**	**Adalimumab**	1.15 (0.45, 3.29)	1.24 (0.33, 4.63)	1.94 (0.70, 5.21)	**0.22 (0.10**, **0.43)**
	0.87 (0.30, 2.21)	**Etanercept**	1.08 (0.30, 3.65)	1.68 (0.65, 4.19)	**0.19 (0.09**, **0.34)**
	0.81 (0.22, 3.01)	0.93 (0.27, 3.34)	**Golimumab**	1.50 (0.42, 5.74)	**0.18 (0.05**, **0.50)**
	0.52 (0.19, 1.43)	0.59 (0.24, 1.54)	0.67 (0.17, 2.36)	**Infliximab**	**0.11 (0.05**, **0.23)**
	**4.63 (2.35**, **9.61)**	**5.37 (2.95**, **10.79)**	**5.67 (2.02**, **18.20)**	**9.15 (4.27**, **18.55)**	**Placebo**
**Serious adverse events**	**Adalimumab**	3.94 (0.27, 74.78)	0.71 (0.03, 25.62)	5.09 (0.25, 179.50)	1.11 (0.14, 10.08)
	0.25 (0.01, 3.74)	**Etanercept**	0.19 (0.01, 4.95)	1.29 (0.07, 36.60)	0.30 (0.04, 1.64)
	1.42 (0.04, 32.86)	5.39 (0.20, 138.42)	**Golimumab**	6.95 (0.23, 304.85)	1.58 (0.10, 18.42)
	0.20 (0.01, 4.08)	0.77 (0.03, 13.83)	0.14 (0.00, 4.43)	**Infliximab**	0.23 (0.02, 1.99)
	0.90 (0.10, 7.19)	3.37 (0.61, 27.38)	0.63 (0.05, 9.84)	4.35 (0.50, 66.28)	**Placebo**
**All cause withdrawals**	**Adalimumab**	1.26 (0.29, 6.97)	1.65 (0.18, 19.51)	3.49 (0.61, 25.53)	1.21 (0.34, 4.58)
	0.79 (0.14, 3.50)	**Etanercept**	1.26 (0.16, 11.48)	2.68 (0.54, 17.02)	0.99 (0.35, 2.28)
	0.61 (0.05, 5.50)	0.80 (0.09, 6.20)	**Golimumab**	2.30 (0.20, 21.53)	0.77 (0.09, 4.44)
	0.29 (0.04, 1.64)	0.37 (0.06, 1.85)	0.44 (0.05, 5.04)	**Infliximab**	0.35 (0.08, 1.25)
	0.83 (0.22, 2.94)	1.01 (0.44, 2.87)	1.30 (0.23, 10.74)	2.82 (0.80, 12.44)	**Placebo**

^*^Comparisons between treatments on the upper left corner should be read from left to right, on the contrary, comparisons on the lower right corner should be read from right to left. Bold values indicate statistical differences.

**Table 4:** Relative ranking of four drugs assessed by SUCRA values.

Treatment	ASAS20 response	ASAS40 response	BASDAI 50% response	SAE	Withdrawals
Adalimumab	0.71	0.57	0.47	0.67	0.71
Etanercept	0.71	0.36	0.54	0.23	0.59
Golimumab	0.44	0.67	0.60	0.75	0.48
Infliximab	0.64	0.88	0.89	0.19	0.12
Placebo	0.00	0.01	0.00	0.66	0.61

## References

[b1] Braun J. & Sieper J. Ankylosing spondylitis. The Lancet 369, 1379–1390 (2007). 10.1016/S0140-6736(07)60635-7 17448825

[b2] Raychaudhuri S. P. & Deodhar A. The classification and diagnostic criteria of ankylosing spondylitis. Journal of Autoimmunity 48–49, 128–133 (2014). 10.1016/j.jaut.2014.01.015 24534717

[b3] Helmick C. G. . Estimates of the prevalence of arthritis and other rheumatic conditions in the United States. Part I. Arthritis Rheum 58, 15–25 (2008). 18163481 10.1002/art.23177

[b4] Khan M. A. Epidemiology of HLA-B27 and arthritis. Clinical rheumatology 15, 10–12 (1996). 8835494 10.1007/BF03342637

[b5] Linden S. V. D., Valkenburg H. A. & Cats A. Evaluation of diagnostic criteria for ankylosing spondylitis. Arthritis & Rheumatism 27, 361–368 (1984). 6231933 10.1002/art.1780270401

[b6] Maxwell L. J. . TNF-alpha inhibitors for ankylosing spondylitis. Cochrane Database Syst Rev 4, CD005468 (2015). 25887212 10.1002/14651858.CD005468.pub2 PMC11200207

[b7] Zochling J. . ASAS/EULAR recommendations for the management of ankylosing spondylitis. Annals of the Rheumatic Diseases 65, 442–452 (2006). 16126791 10.1136/ard.2005.041137 PMC1798102

[b8] Sieper J. & Braun J. Management of ankylosing spondylitis. In: Ankylosing Spondylitis. Springer, p 49–72 (2011).

[b9] Knobloch K., Yoon U. & Vogt P. M. Preferred reporting items for systematic reviews and meta-analyses (PRISMA) statement and publication bias. J Craniomaxillofac Surg 39, 91–92 (2011). 21145753 10.1016/j.jcms.2010.11.001

[b10] van der Linden S., Valkenburg H. A. & Cats A. Evaluation of diagnostic criteria for ankylosing spondylitis. A proposal for modification of the New York criteria. Arthritis Rheum 27, 361–368 (1984). 6231933 10.1002/art.1780270401

[b11] Higgins J. P. T. & Green S. Cochrane Handbook for Systematic Reviews of Interventions. Version 5.1.0. The Cochrane Collaboration (2011).

[b12] Salanti G., Ades A. E. & Ioannidis J. P. Graphical methods and numerical summaries for presenting results from multiple-treatment meta-analysis: an overview and tutorial. J Clin Epidemiol 64, 163–171 (2011). 20688472 10.1016/j.jclinepi.2010.03.016

[b13] Huang F. . Efficacy and safety of adalimumab in Chinese adults with active ankylosing spondylitis: results of a randomised, controlled trial. Ann Rheum Dis 73, 587–594 (2014). 23475983 10.1136/annrheumdis-2012-202533

[b14] Bao C. . Safety and efficacy of golimumab in Chinese patients with active ankylosing spondylitis: 1-year results of a multicentre, randomized, double-blind, placebo-controlled phase III trial. Rheumatology (Oxford) 53, 1654–1663 (2014). 24729398 10.1093/rheumatology/keu132

[b15] Dougados M. . Efficacy of etanercept on rheumatic signs and pulmonary function tests in advanced ankylosing spondylitis: results of a randomised double-blind placebo-controlled study (SPINE). Ann Rheum Dis 70, 799–804 (2011). 21317434 10.1136/ard.2010.139261 PMC3070274

[b16] Inman R. D. & Maksymowych W. P. A double-blind, placebo-controlled trial of low dose infliximab in ankylosing spondylitis. J Rheumatol 37, 1203–1210 (2010). 20231198 10.3899/jrheum.091042

[b17] Dijkmans B. . Etanercept in the longterm treatment of patients with ankylosing spondylitis. J Rheumatol 36, 1256–1264 (2009). 19411393 10.3899/jrheum.081033

[b18] Inman R. D. . Efficacy and safety of golimumab in patients with ankylosing spondylitis: results of a randomized, double-blind, placebo-controlled, phase III trial. Arthritis Rheum 58, 3402–3412 (2008). 18975305 10.1002/art.23969

[b19] van der Heijde D. . Efficacy and safety of adalimumab in patients with ankylosing spondylitis: results of a multicenter, randomized, double-blind, placebo-controlled trial. Arthritis Rheum 54, 2136–2146 (2006). 16802350 10.1002/art.21913

[b20] van der Heijde D. . Etanercept 50 mg once weekly is as effective as 25 mg twice weekly in patients with ankylosing spondylitis. Ann Rheum Dis 65, 1572–1577 (2006). 16968715 10.1136/ard.2006.056747 PMC1798458

[b21] van der Heijde D. . Efficacy and safety of infliximab in patients with ankylosing spondylitis: results of a randomized, placebo-controlled trial (ASSERT). Arthritis Rheum 52, 582–591 (2005). 15692973 10.1002/art.20852

[b22] Marzo-Ortega H. . Infliximab in combination with methotrexate in active ankylosing spondylitis: a clinical and imaging study. Ann Rheum Dis 64, 1568–1575 (2005). 15829577 10.1136/ard.2004.022582 PMC1755262

[b23] Calin A. . Outcomes of a multicentre randomised clinical trial of etanercept to treat ankylosing spondylitis. Ann Rheum Dis 63, 1594–1600 (2004). 15345498 10.1136/ard.2004.020875 PMC1754832

[b24] Brandt J. . Development and preselection of criteria for short term improvement after anti-TNF alpha treatment in ankylosing spondylitis. Ann Rheum Dis 63, 1438–1444 (2004). 15044211 10.1136/ard.2003.016717 PMC1754796

[b25] Davis J. C. Jr. . Recombinant human tumor necrosis factor receptor (etanercept) for treating ankylosing spondylitis: a randomized, controlled trial. Arthritis Rheum 48, 3230–3236 (2003). 14613288 10.1002/art.11325

[b26] Brandt J. . Six-month results of a double-blind, placebo-controlled trial of etanercept treatment in patients with active ankylosing spondylitis. Arthritis Rheum 48, 1667–1675 (2003). 12794835 10.1002/art.11017

[b27] Gorman J. D., Sack K. E. & Davis J. C. Jr. Treatment of ankylosing spondylitis by inhibition of tumor necrosis factor alpha. N Engl J Med 346, 1349–1356 (2002). 11986408 10.1056/NEJMoa012664

[b28] Braun J. . Treatment of active ankylosing spondylitis with infliximab: a randomised controlled multicentre trial. Lancet 359, 1187–1193 (2002). 11955536 10.1016/s0140-6736(02)08215-6

[b29] Choy E. H. & Panayi G. S. Cytokine pathways and joint inflammation in rheumatoid arthritis. N Engl J Med 344, 907–916 (2001). 11259725 10.1056/NEJM200103223441207

[b30] Braun J. . Use of immunohistologic and *in situ* hybridization techniques in the examination of sacroiliac joint biopsy specimens from patients with ankylosing spondylitis. Arthritis & Rheumatism 38, 499–505 (1995). 7718003 10.1002/art.1780380407

[b31] Weinblatt M. E. . Adalimumab, a fully human anti–tumor necrosis factor α monoclonal antibody, for the treatment of rheumatoid arthritis in patients taking concomitant methotrexate: the ARMADA trial. Arthritis & Rheumatism 48, 35–45 (2003). 12528101 10.1002/art.10697

[b32] Keystone E. C. . Golimumab, a human antibody to TNF-α given by monthly subcutaneous injections, in active rheumatoid arthritis despite methotrexate: the GO-FORWARD Study. Annals of the rheumatic diseases (2008). 10.1136/ard.2008.099010 PMC2674549 19066176

[b33] Weinblatt M. E. . A trial of etanercept, a recombinant tumor necrosis factor receptor: Fc fusion protein, in patients with rheumatoid arthritis receiving methotrexate. New England Journal of Medicine 340, 253–259 (1999). 9920948 10.1056/NEJM199901283400401

[b34] Maini R. . Infliximab (chimeric anti-tumour necrosis factor α monoclonal antibody) versus placebo in rheumatoid arthritis patients receiving concomitant methotrexate: a randomised phase III trial. The Lancet 354, 1932–1939 (1999). 10.1016/s0140-6736(99)05246-0 10622295

