# Supplementary material for: Efficacy and safety of TNF-α inhibitors for active ankylosing spondylitis patients: Multiple treatment comparisons in a network meta-analysis
Source: Sci Rep. 2016 Sep 26;6:32768. doi: 10.1038/srep32768 (PMC5036083; doi:10.1038/srep32768)
Supplement: Supplementary Information [file srep32768-s1.pdf]

Efficacy and safety of TNF-α inhibitors for active ankylosing spondylitis patients: Multiple treatment comparisons in a network meta-analysis

Running title: TNF-α inhibitors and active AS

Wei Liu[1*], Yuan-hao Wu[1,2*], Lei Zhang[1], Xiao-ya Liu[1], Bin Xue[1], Bin Liu[1], Yi Wang[1], Yang Ji[3]

[1]Department of Rheumatology and Immunology, The First Teaching Hospital of Tianjin University of Traditional Chinese Medicine, Tianjin, China

[2]The Institute of Basic Research in Clinical Medicine, China Academy of Chinese Medical Science

[3]The 272nd Hospital of Chinese People's Liberation Army, Tianjin, China

[*]**Correspondence should be addressed to** Wei Liu, Department of Rheumatology and Immunology, The First Teaching Hospital of Tianjin University of Traditional Chinese Medicine, An-shan-xi Road #314, Nankai District, Tianjin,300193, China (e-mail: liuwei_tcm@163.com; tel.: +86 022-27986638) or Yuan-hao Wu, Department of Rheumatology and Immunology, The First Teaching Hospital of Tianjin University of Traditional Chinese Medicine, An-shan-xi Road #314, Nankai District, Tianjin,300193, China; The Institute of Basic Research in Clinical Medicine, China Academy of Chinese Medical Science (e-mail: yuanhaowu@yeah.net)

Wei Liu and Yuan-hao Wu contributed equally as the first co-authors for the work of literature search, data extraction and writing.

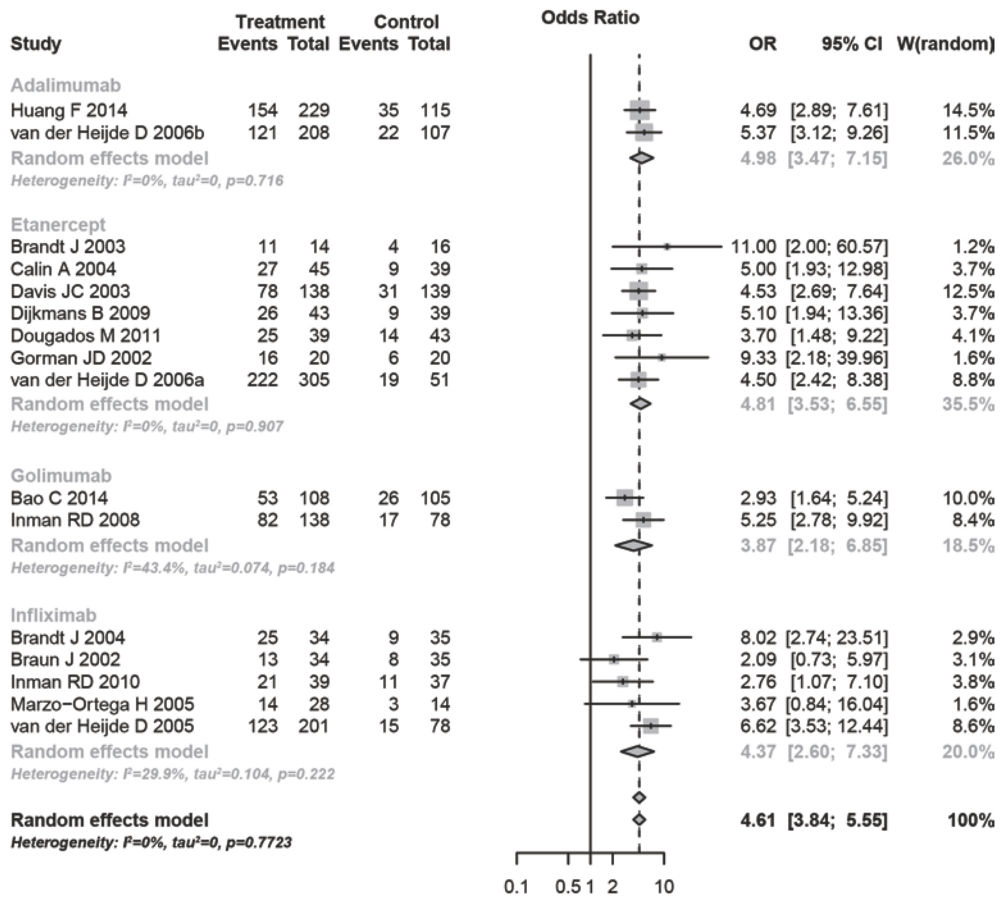

Figure S1. Forest plots of four TNF-α inhibitors for ASAS 20 response. The OR values from each study are represented by squares, and the confidence intervals (CIs) are indicated by error bars. The rhombus indicates whether the pooled OR value is under the fixed effect or random effects model.

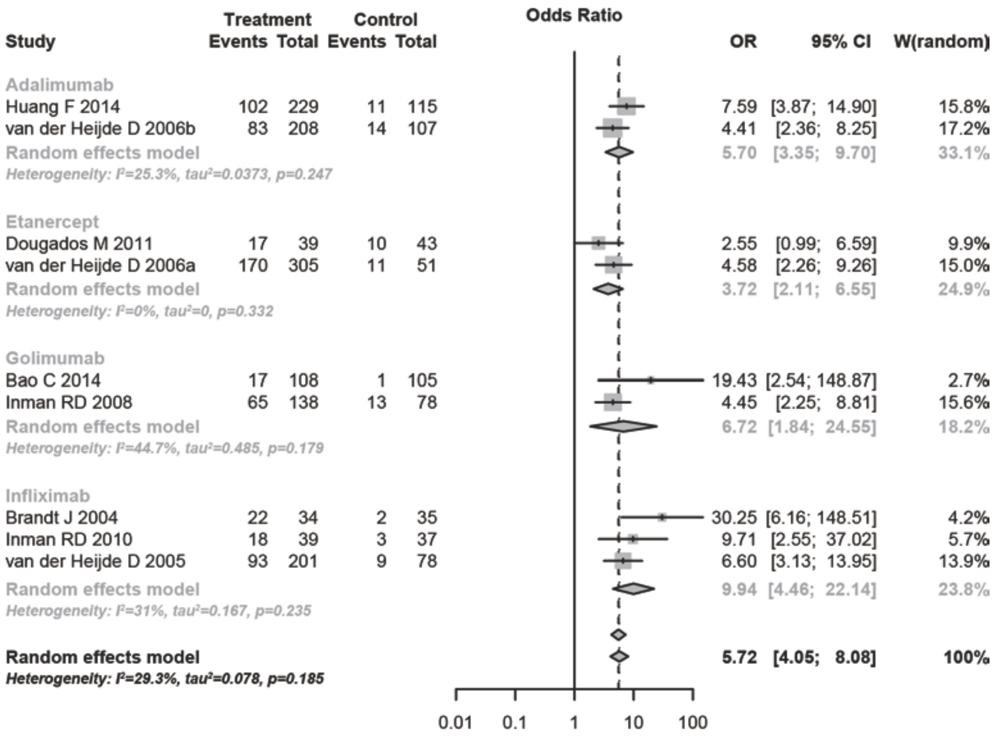

Figure S2. Forest plots of four TNF-α inhibitors for ASAS 40 response. The OR values from each study are represented by squares, and the confidence intervals (CIs) are indicated by error bars. The rhombus indicates whether the pooled OR value is under random effects model.

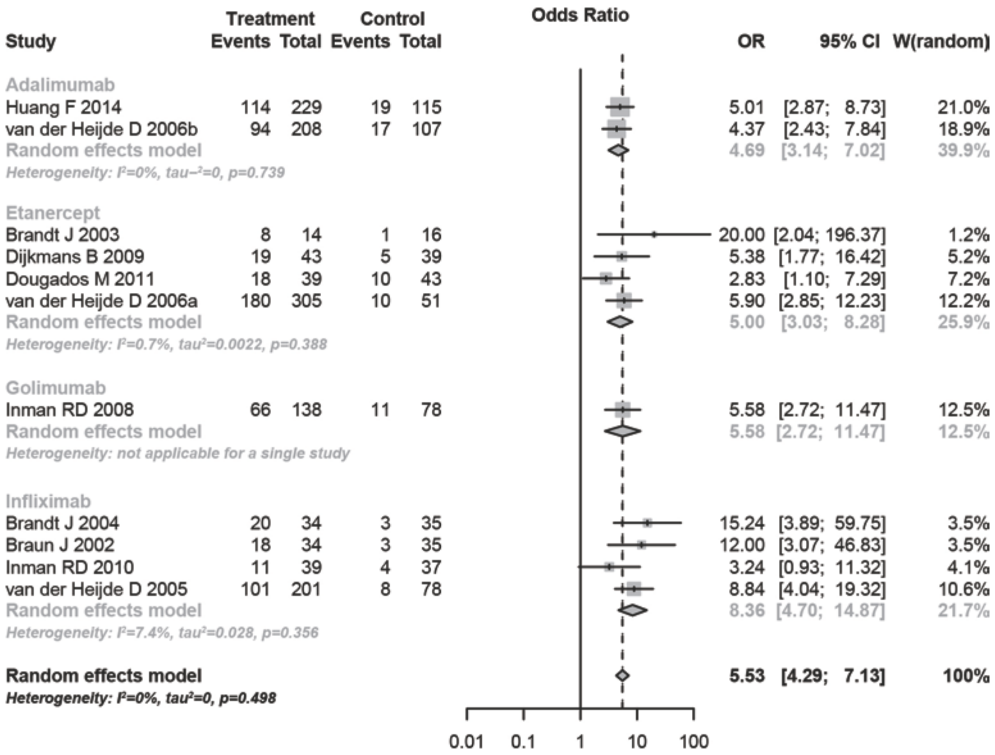

Figure S3. Forest plots of four TNF-α inhibitors for BASDAI 50%. The OR values from each study are represented by squares, and the confidence intervals (CIs) are indicated by error bars. The rhombus indicates whether the pooled OR value is under random effects model.

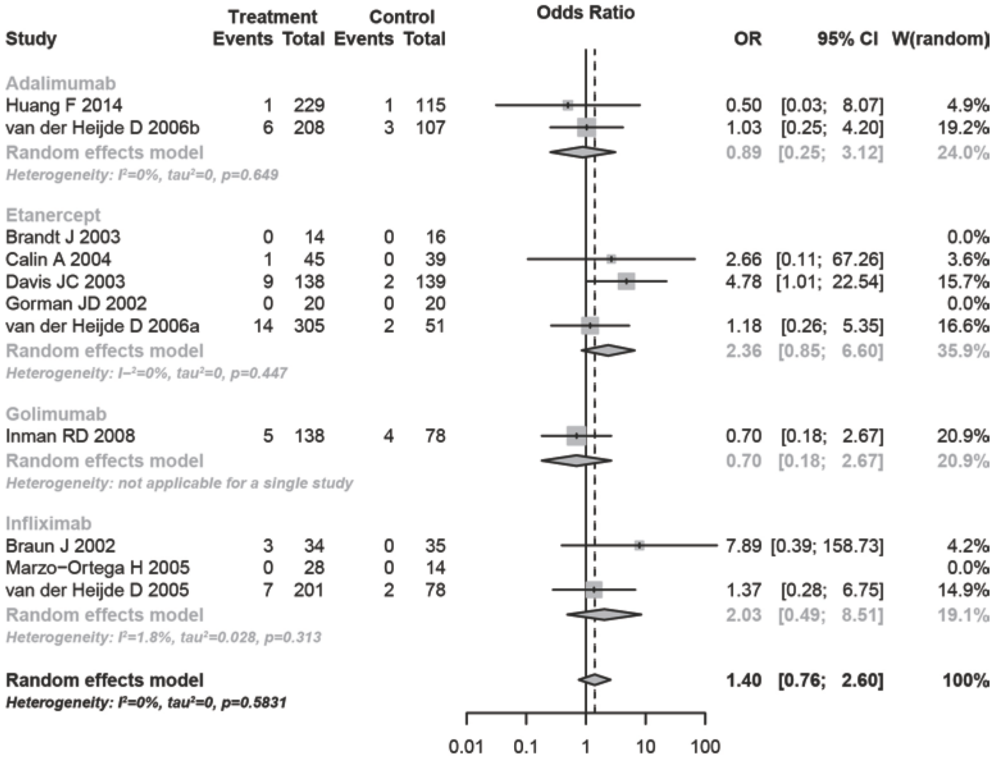

Figure S4. Forest plots of four TNF-α inhibitors for serious adverse events. The OR values from each study are represented by squares, and the confidence intervals (CIs) are indicated by error bars. The rhombus indicates whether the pooled OR value is under random effects model.

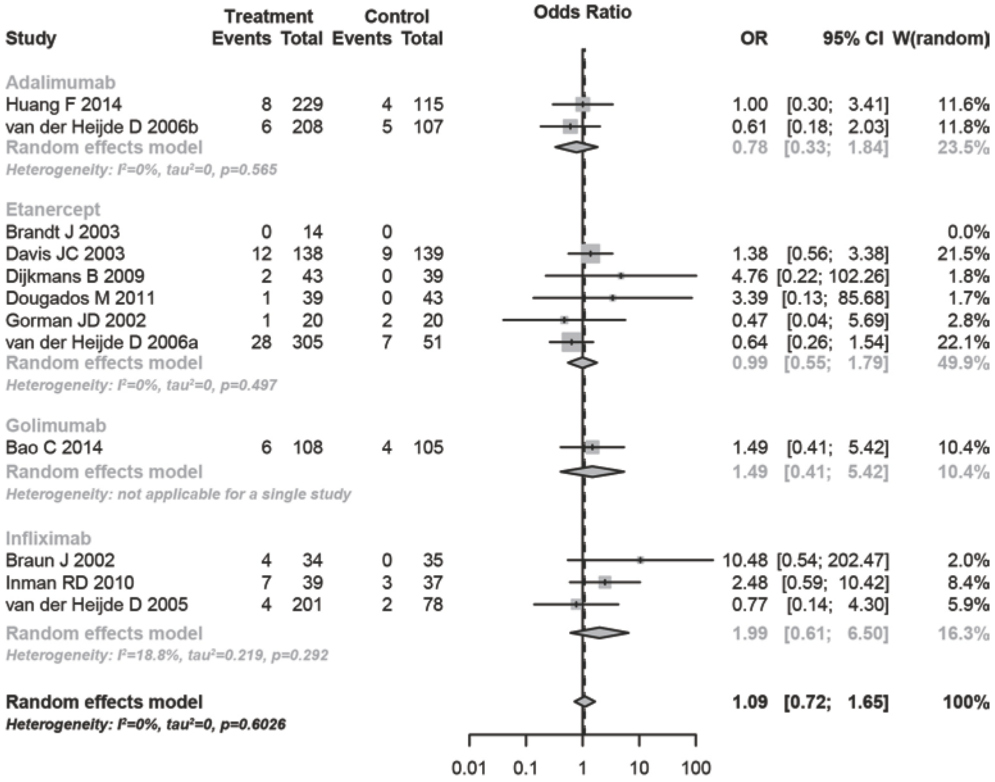

Figure S5. Forest plots of four TNF-α inhibitors for all cause withdrawals. The OR values from each study are represented by squares, and the confidence intervals (CIs) are indicated by error bars. The rhombus indicates whether the pooled OR value is under random effects model.

Table S1 The detailed research strategy of each database.

**PubMed**	**(1) AS**
	"Spondylitis, Ankylosing"[Mesh] OR Ankylosing Spondylitis[tiab] OR Spondyloarthritis Ankylopoietica[tiab] OR Ankylosing Spondylarthritis[tiab] OR Ankylosing Spondylarthritides[tiab] OR Ankylosing Spondylitis[tiab] OR Spondylarthritis Ankylopoietica[tiab] OR Bechterew Disease[tiab] OR Bechterew's Disease[tiab] OR Bechterews Disease[tiab] OR Marie-Struempell Disease[tiab] OR Marie Struempell Disease[tiab] OR Rheumatoid Spondylitis[tiab] OR Spondylitis Ankylopoietica[tiab] OR Ankylosing Spondyloarthritis[tiab] OR Ankylosing Spondyloarthritides[tiab]
	**(2) Drugs**
	Anti-TNF-alpha agents[tiab] OR Tumor necrosis factor-alpha inhibitor[tiab] OR TNF blockers[tiab] OR TNF inhibitors[tiab] OR "Adalimumab"[Mesh] OR adalimumab[tiab] OR "Etanercept"[Mesh] OR etanercept[tiab] OR "Infliximab"[Mesh] OR infliximab[tiab] OR "golimumab"[Supplementary Concept] OR golimumab[tiab]
	**(3) RCT**
	"randomized controlled trial"[pt] OR "controlled clinical trial"[pt] OR "Controlled Clinical Trials as Topic"[mh] OR "randomized controlled trials as topic"[mh] OR randomized[tiab] OR placebo[tiab] OR "clinical trials as topic"[mh] OR "controlled clinical trials as topic"[mh] OR "random allocation"[mh] OR randomization[tiab] OR randomly[tiab] OR random[tiab] OR ((double* [tw] OR trebl* [tw] OR tripl* [tw]) AND (mask* [tw] OR blind* [tw]))
**Embase**	**(1) AS**
	ankylosing spondylitis'/exp OR 'Spondyloarthritis Ankylopoietica':ti,ab OR 'Ankylosing Spondylarthritis':ti,ab OR 'Ankylosing Spondylarthritides':ti,ab OR 'Ankylosing Spondylitis':ti,ab OR 'Spondylarthritis Ankylopoietica':ti,ab OR 'Bechterew Disease':ti,ab OR 'Bechterew's Disease':ti,ab OR 'Bechterews Disease':ti,ab OR 'Marie-Struempell Disease':ti,ab OR 'Marie Struempell Disease':ti,ab OR 'Rheumatoid Spondylitis':ti,ab OR 'Spondylitis Ankylopoietica':ti,ab OR 'Ankylosing Spondyloarthritis':ti,ab OR 'Ankylosing Spondyloarthritides':ti,ab OR 'ankylopoietic spondylarthritis':ti,ab OR 'ankylopoietic spondylitis':ti,ab OR 'ankylosing spine':ti,ab OR 'ankylosing spondilitis':ti,ab OR 'ankylosing spondylarthrosis':ti,ab OR 'ankylosis spondylitis':ti,ab OR 'ankylotic spondylitis':ti,ab OR

	'bekhterev disease':ti,ab OR 'spinal ankylosis':ti,ab OR 'spine ankylosis':ti,ab OR 'spondylarthritis ankylosans':ti,ab OR 'spondylarthrosis ankylopoietica':ti,ab OR 'spondylitis ankylopoetica; spondyloarthritis ankylopoietica':ti,ab OR 'vertebral ankylosis':ti,ab
	**(2) Drugs**
	tumor necrosis factor alpha inhibitor'/exp OR 'tumour necrosis factor alpha inhibitor':ti,ab OR 'anti TNF alpha agent':ti,ab OR 'TNF alpha inhibitor':ti,ab OR 'anti-TNF alpha':ab,ti OR 'anti-tumor necrosis factor alpha':ab,ti OR 'anti-TNF':ab,ti OR 'adalimumab'/exp OR adalimumab:ab,ti OR 'etanercept'/exp OR etanercept:ab,ti OR 'infliximab'/exp OR infliximab:ab,ti OR 'golimumab'/exp or golimumab:ab,ti
	(3) RCT
	random*:ab,ti OR placebo*:ab,ti OR 'double blind procedure'/exp OR 'triple blind procedure'/exp OR 'randomized controlled trial'/exp OR 'controlled clinical trial'/exp OR 'crossover procedure'/exp OR 'random allocation':ab,ti OR placebo:ab,ti OR 'randomized controlled trial':ab,ti OR 'randomised controlled trial':ab,ti OR rct:ab,ti OR 'randomly allocated':ab,ti OR 'allocated randomly':ab,ti OR ((double:ab,ti OR treble:ab,ti OR triple:ab,ti) AND (blind:ab,ti OR mask:ab,ti))
**CNKI**	**(1) AS**
	TI=强直性脊柱炎 OR AB=强直性脊柱炎 OR TI=类风湿性脊柱炎 OR AB=类风湿性脊柱炎 OR TI=变形性脊柱炎 OR AB=变形性脊柱炎
	**(2) Drugs**
	TI=TNF-alpha OR AB=TNF-alpha OR TI=TNF-a OR AB=TNF-a OR TI=阿达木单抗 OR TI=依那西普 OR TI=高利单抗 OR TI=英夫利昔 OR AB=阿达木单抗 OR AB=依那西普 OR AB=高利单抗 OR AB=英夫利昔

Table S2 Jadad Scale.

First author, year	Randomization	Blinding	Withdraw
Bao C, 2014	2	2	1
Brandt J, 2003	2	2	1
Brandt J, 2004	2	2	1
Braun J, 2002	2	0	1
Calin A, 2004	2	2	1
Davis JC, 2003	2	2	1
Dijkmans B, 2009	2	2	1
Dougados M, 2011	2	2	1
Gorman JD, 2002	2	2	1
Huang F, 2014	2	2	0
Inman RD, 2008	2	2	1
Inman RD, 2010	2	2	1
Marzo-Ortega H, 2005	2	2	1
van der Heijde D, 2005	2	2	1
van der Heijde D, 2006a	2	2	1
